# Supplementary material for: The cholesterol transporter NPC1 is essential for epigenetic regulation and maturation of oligodendrocyte lineage cells
Source: Nat Commun. 2023 Jul 5;14:3964. doi: 10.1038/s41467-023-39733-6 (PMC10322873; doi:10.1038/s41467-023-39733-6)
Supplement: Supplementary file 1 — Supplementary Information [file 41467_2023_39733_MOESM1_ESM.pdf]

Supplementary Information

The cholesterol transporter NPC1 is essential for epigenetic regulation and maturation of oligodendrocyte lineage cells

Thaddeus J. Kunkel[1,#], Alice Townsend[2,#], Kyle A. Sullivan[3], Jean Merlet[2], Edward H Schuchman[4], Daniel A. Jacobson[3], Andrew P. Lieberman[1,*]

[1]Department of Pathology, University of Michigan Medical School, Ann Arbor, MI, USA

[2]The Bredesen Center for Interdisciplinary Research and Graduate Education, University of Tennessee Knoxville, Knoxville, TN, USA

[3]Computational and Predictive Biology, Oak Ridge National Laboratory, Oak Ridge, TN, USA

[4]Department of Genetics and Genomic Sciences, Mount Sinai School of Medicine, New York, NY USA

[#]These authors contributed equally to this work.

*Address correspondence and requests to:
Department of Pathology
University of Michigan Medical School
3510 MSRB1, 1150 W. Medical Center Dr.
Ann Arbor, MI 48109
Telephone: (734) 647-4624
Email: liebermn@umich.edu

The authors have declared that no conflict of interest exists.

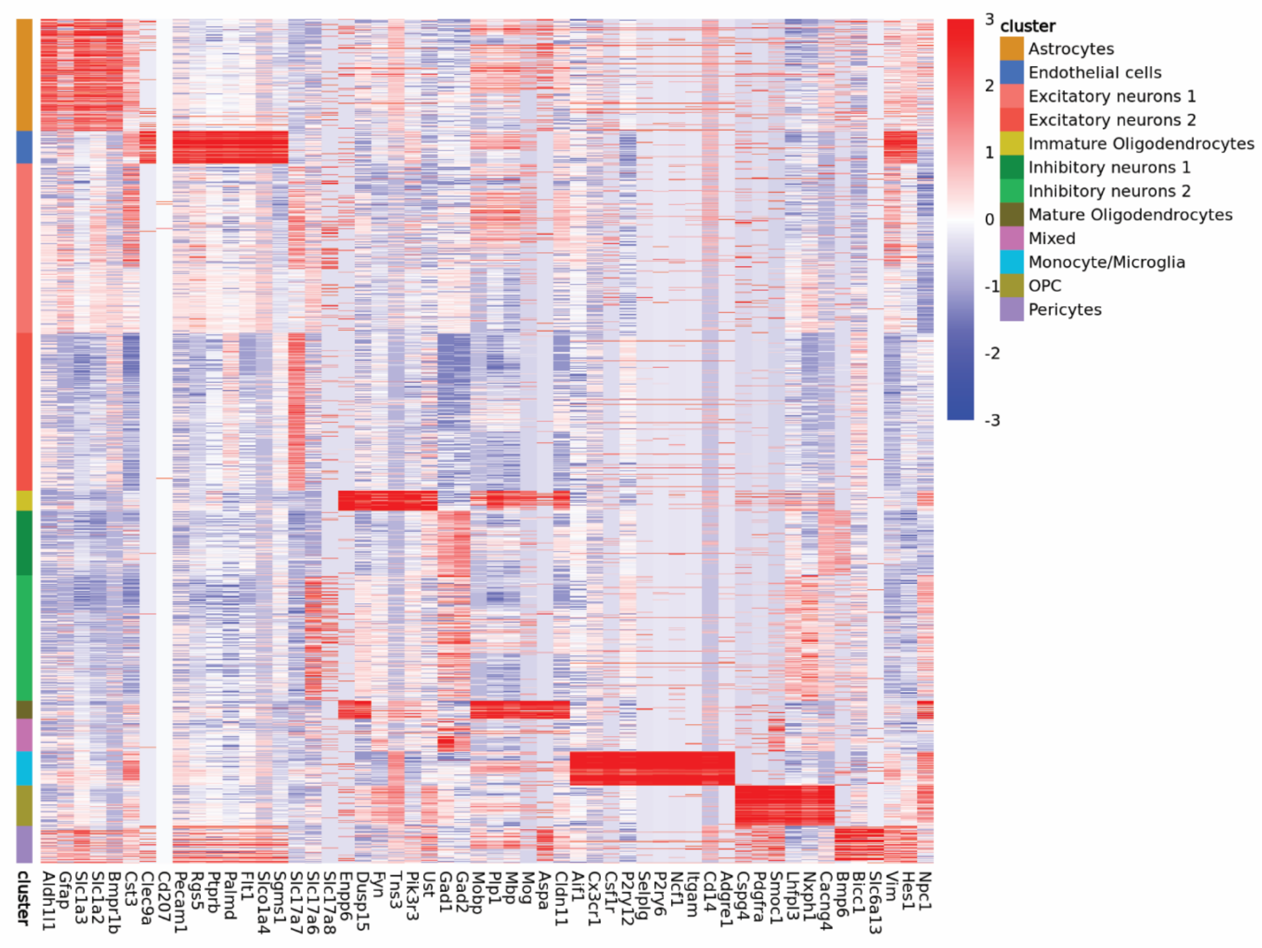

Supplementary Figure 1. Heatmap of marker gene expression. Heatmap showing the expression pattern of marker genes used to identify cell clusters. Genes are listed across the bottom. Colors to the left of the heatmap indicate cell clusters.

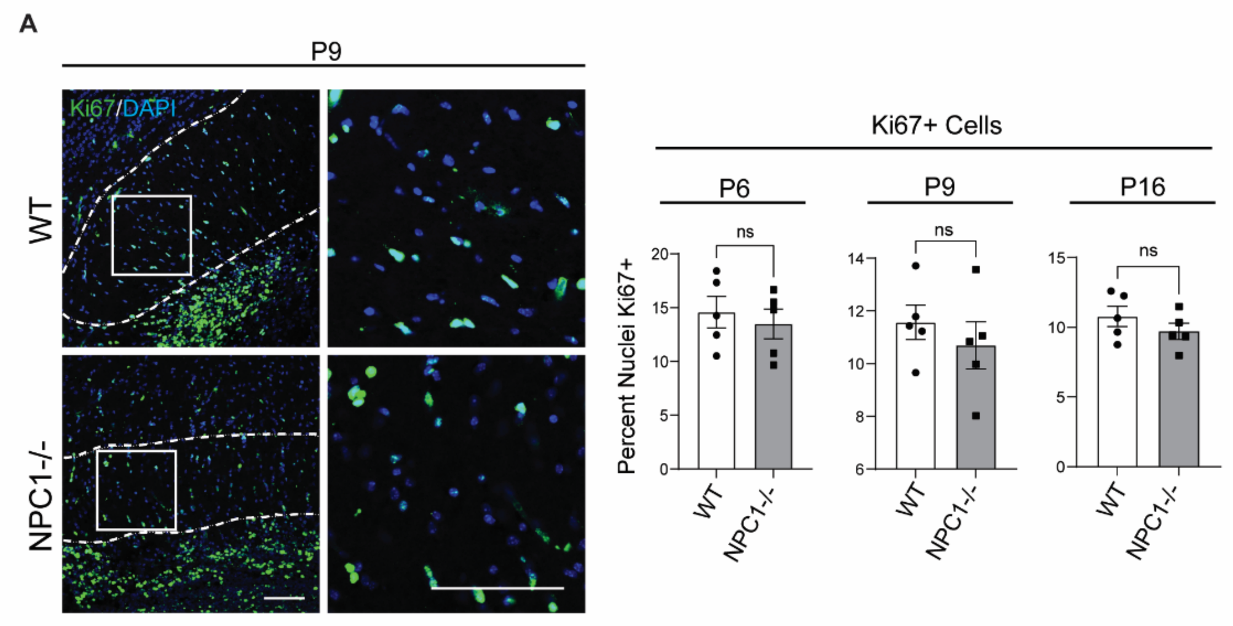

Supplementary Figure 2. Cell proliferation in corpus callosum. Ki67 staining was performed on brains from P6, P9, and P16 WT and *Npc1*$^{-/-}$ mice to assess cell proliferation. Ki67 in green; nuclei stained by DAPI (blue). Dashed lines indicate corpus callosum. Scale bar = 100 µm. Quantified at right. Data are mean ± SEM. n.s. = not significant by two-tailed Student's t-test. n=5; t=0.5520, 0.7868, 1.115; df=8; p=0.5960, 0.4541, 0.2973. Source data are provided as a Source Data file.

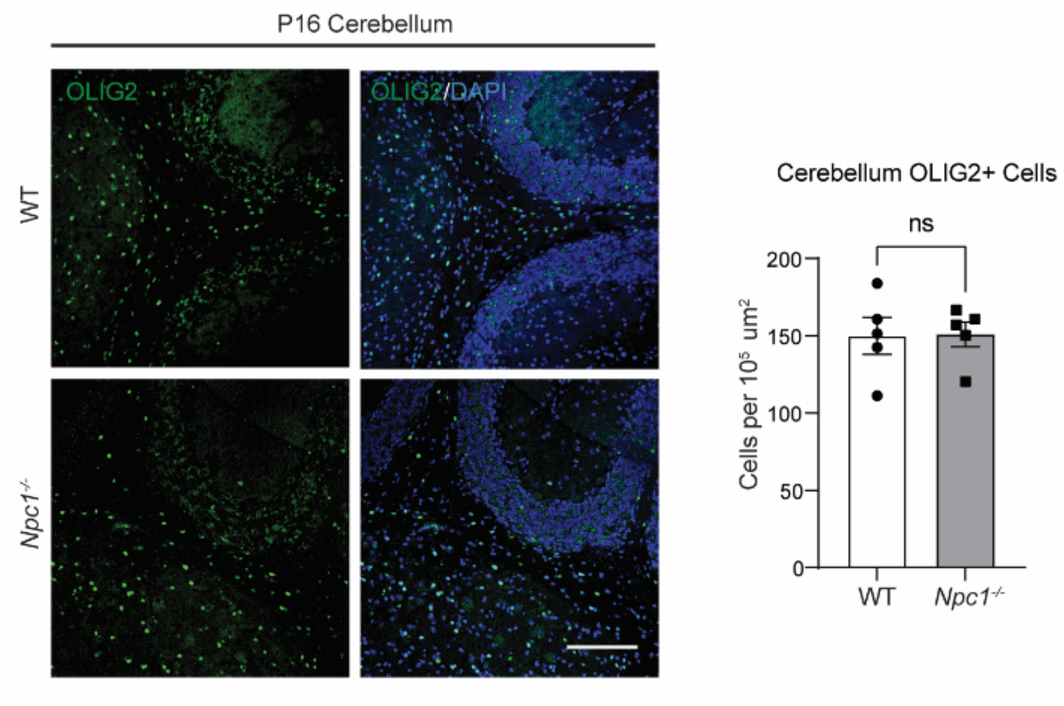

Supplementary Figure 3. OLIG2-positive cells in *Npc1*$^{-/-}$ cerebellum. OLIG2 staining was performed on sagittal brain sections from P16 mice. Images were taken from cerebellar white matter and OLIG2+ cells (green) were quantified. Nuclei stained by DAPI (blue). Scale bar = 150 μm. Images quantified at right. Data are mean ± SEM. n.s. = not significant by two-tailed Student's t-test. n=5 mice; t=0.2055; df=8; p=0.8423. Source data are provided as a Source Data file.

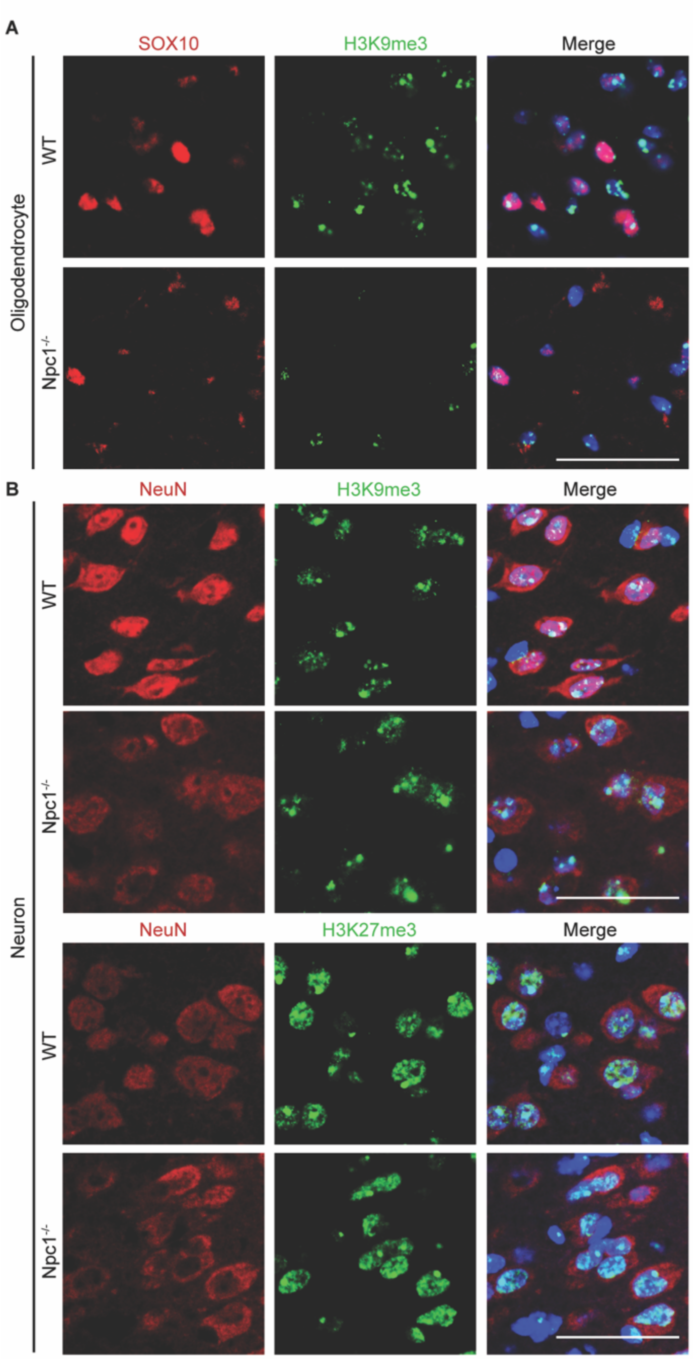

Supplementary Figure 4. Histone methylation staining. Brain sections from P16 WT and *Npc1*$^{-/-}$ mice were stained for H3K9me3 and H3K27me3 (green). Sections were co-stained for SOX10 (oligodendrocytes, visualized in corpus callosum) or NeuN (neurons, visualized in cortex) (red). Nuclei stained with DAPI (blue). Scale bar = 50 μm. These

experiments were performed on 5 biological replicates. Quantifications are shown in Figure 4B.

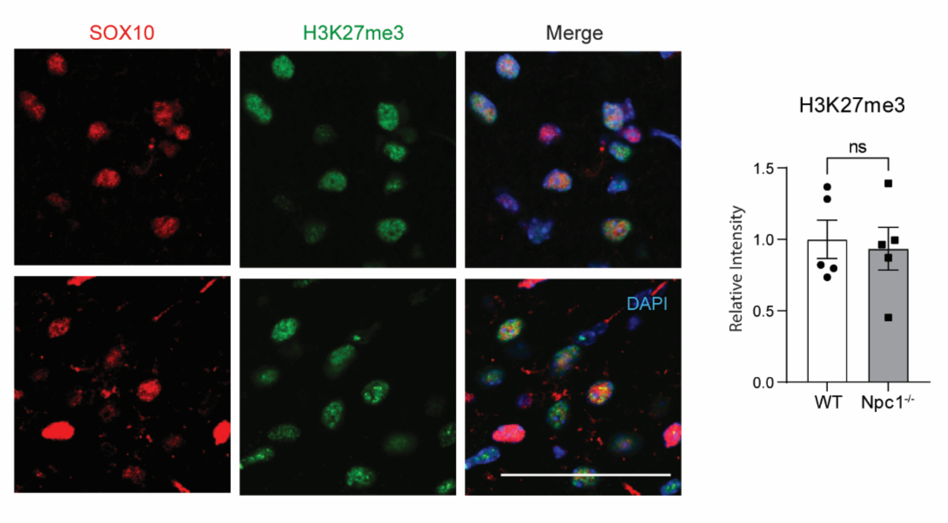

Supplementary Figure 5. H3K27me3 staining in cerebellum. H3K27me3 staining (green) was performed on sagittal brain sections from WT and *Npc1*$^{-/-}$ mice at P16. Sections were co-stained for SOX10 (red) and DAPI (blue). Images were taken from cerebellar white matter and H3K27me3 intensity within SOX10+ cells was quantified relative to WT. Scale bar = 50 μm. Data are mean ± SEM. n.s. = not significant, by two-tailed Student's t-test. n=5 mice; t=0.3226; df=8; p=0.7523. Source data are provided as a Source Data file.

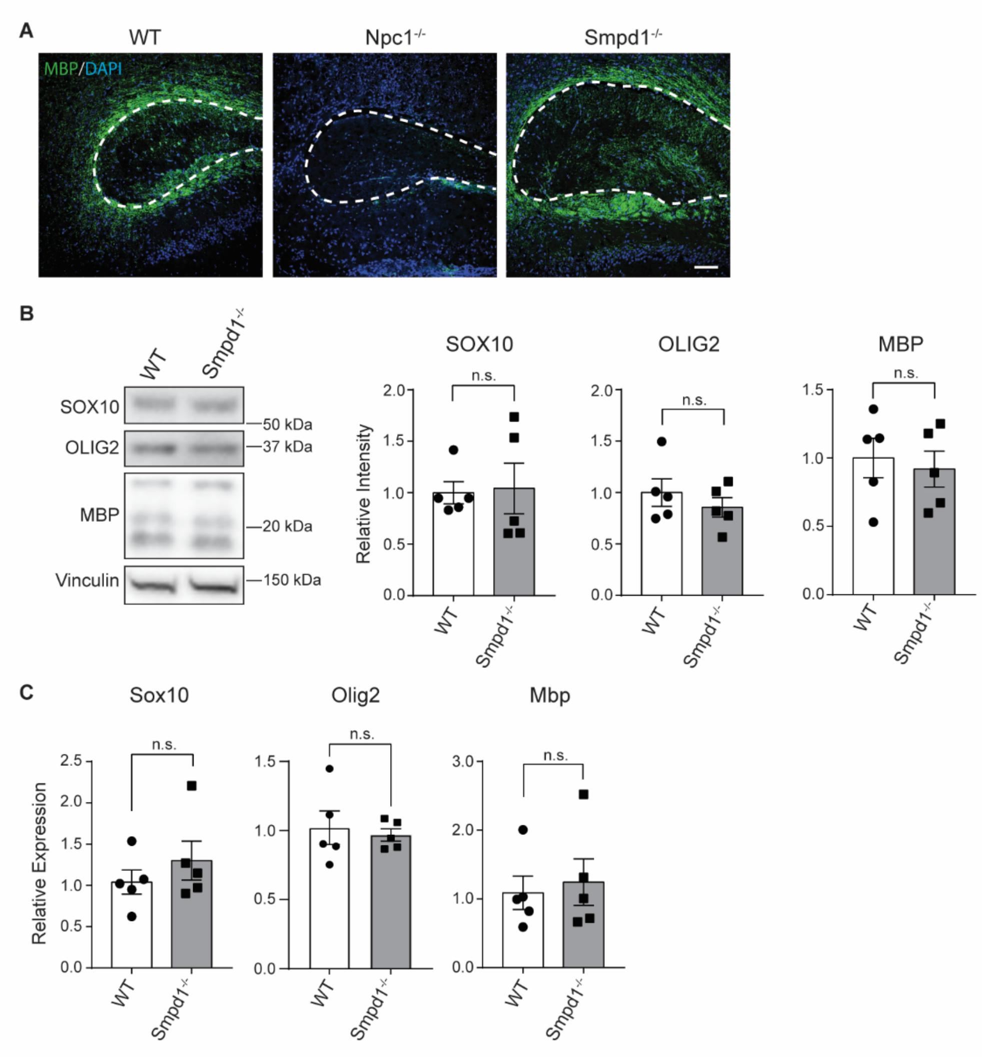

Supplementary Figure 6. *Smpd1$^{-/-}$* mice do not display developmental hypomyelination. Brains from WT and *Smpd1$^{-/-}$* mice at P16 were analyzed for myelination. (A) Midline sagittal sections were stained for MPB (green). Nuclei stained blue. Corpus callosum indicated by dashed line. This experiment was done on 3 biological replicates per group, all with similar results. Scale bar = 100 μm. (B) Western blot for SOX10, OLIG2, and MBP. Quantified at right. (C) Relative expression of Mbp, Sox10, and Olig2 quantifed by qRT-PCR. Data are mean ± SEM. n.s. = not significant by two-tailed Student's t-test. (B) n=5 mice; t=0.1570, 0.8786, 0.4145; df=8; p=0.8791,

0.4053, 0.6894; (C) n=5 mice; t=0.94, 0.41, 0.37; df=8; p=0.3768, 0.6930, 0.7181.

Source data are provided as a Source Data file.

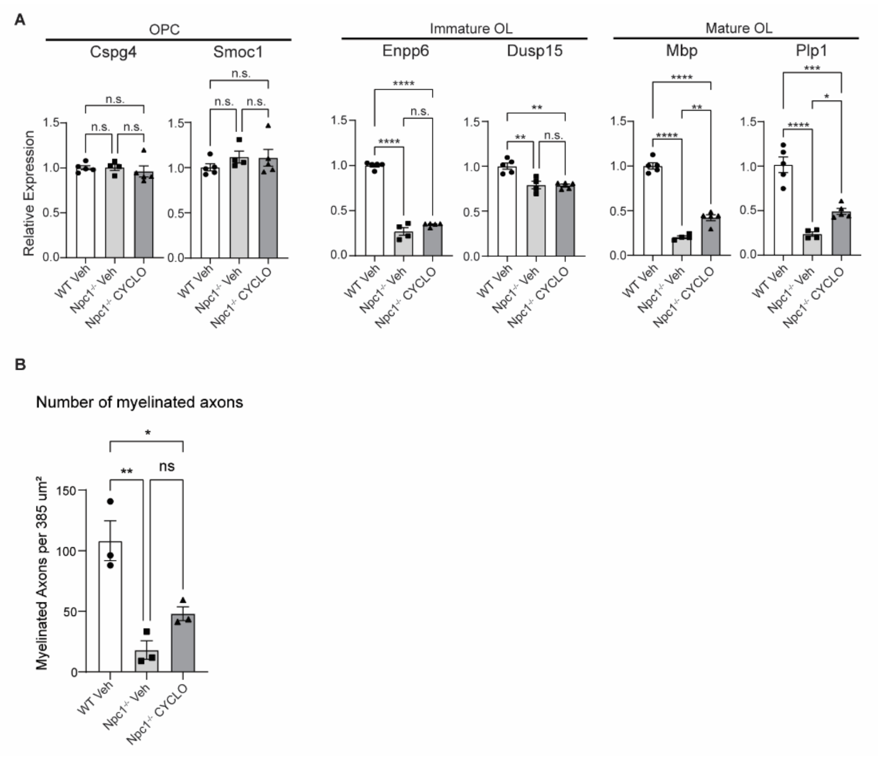

Supplementary Figure 7. Improved myelination after 2-hydroxypropyl-β-cyclodextrin treatment. Mice were administered 2-hydroxypropyl-β-cyclodextrin (4000mg/kg, i.p.) or a vehicle control (saline) at P7. (A) Brains were collected at P16 and qRT-PCR was performed for genes associated with specific stages of the oligodendrocyte lineage. (B) Brains from P16 mice were sectioned for TEM and the number of myelinated axons was quantified. Data are mean ± SEM. n.s. = not significant; *p<0.05, **p<0.005, ***p<0.001, ****p<0.0001 by one-way ANOVA. (A) n=5, 4, 5 mice for each group; F=0.32, 0.82, 295.5, 15.97, 177.0, 43.84; df=2; p=0.7307, 0.4667, <0.0001, 0.0006, <0.0001, <0.0001; (B) n=3 mice; F=17.57; df=2; p=**0.0027, *0.0190, 0.2099. Source data are provided as a Source Data file.

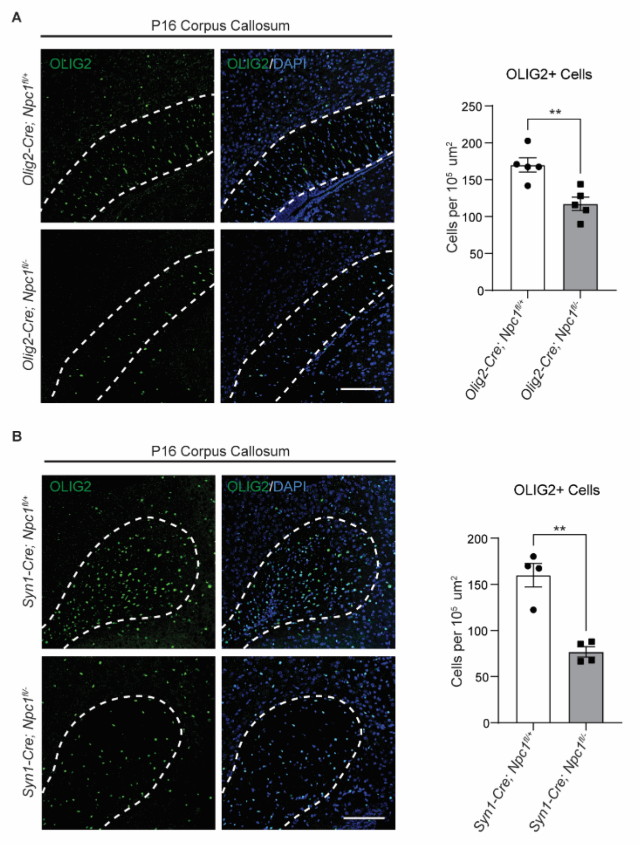

Supplementary Figure 8. OLIG2-positive cells in Cre lines. OLIG2 staining was performed on sagittal brain sections from P16 mice. OLIG2+ cells (green) were quantified in the corpus callosum from *Olig2-Cre;Npc1*$^{fl/+}$ and *Olig2-Cre;Npc1*$^{fl/-}$ (A) and *Syn1-Cre;Npc1*$^{fl/+}$ and *Syn-Cre;Npc1*$^{fl/-}$ mice (B). Nuclei stained by DAPI (blue). Dashed lines indicate corpus callosum. Scale bars = 150 µm. Images quantified at right. Data are mean ± SEM. **p<0.005 by two-tailed Student's t-test. (A) n=5 mice; t=2.554; df=8; p=0.0340; (B) n=4 mice; t=5.931; df=6; p=0.0010. Source data are provided as a Source Data file.

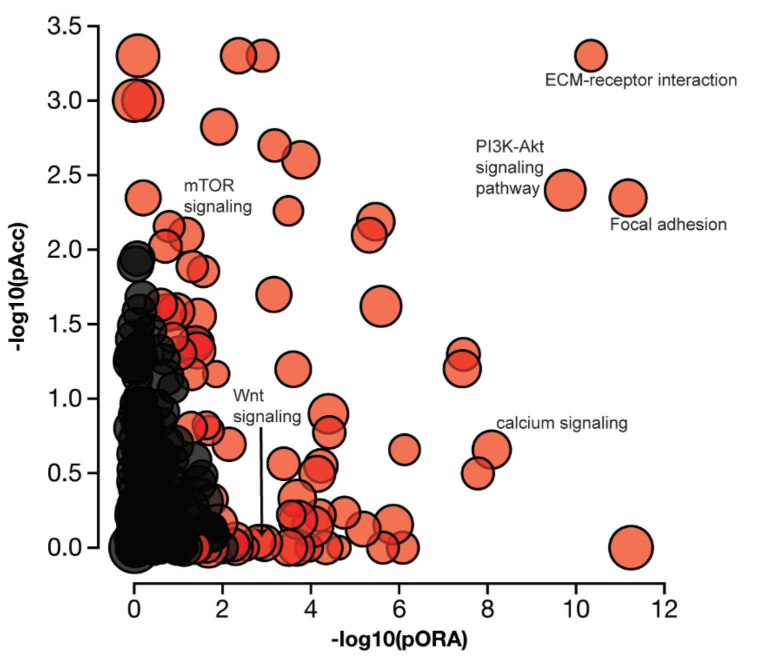

Supplementary Figure 9. Pathway analysis on O4+ cells. RNA-seq was performed on O4-puritied cells from WT and *Npc1*$^{-/-}$ mice at P16. Gene expression changes were determined using DESeq2 and analyzed using Impact analysis with a false discovery rate (FDR) correction from iPathwayGuide (Advaita Corporation), which considers over-representation of DE genes as well as the topology of the pathway. This plot shows the pathways that are enriched. The x-axis represents the p-values obtained using over-representation analysis, while the y-axis is the p-value obtained from total perturbation accumulation in the pathway. Each dot represents a pathway, with dot size correlating with number of genes present from each pathway. Red indicates a significant enrichment (adjusted p-value<0.05). Select pathways are labeled.

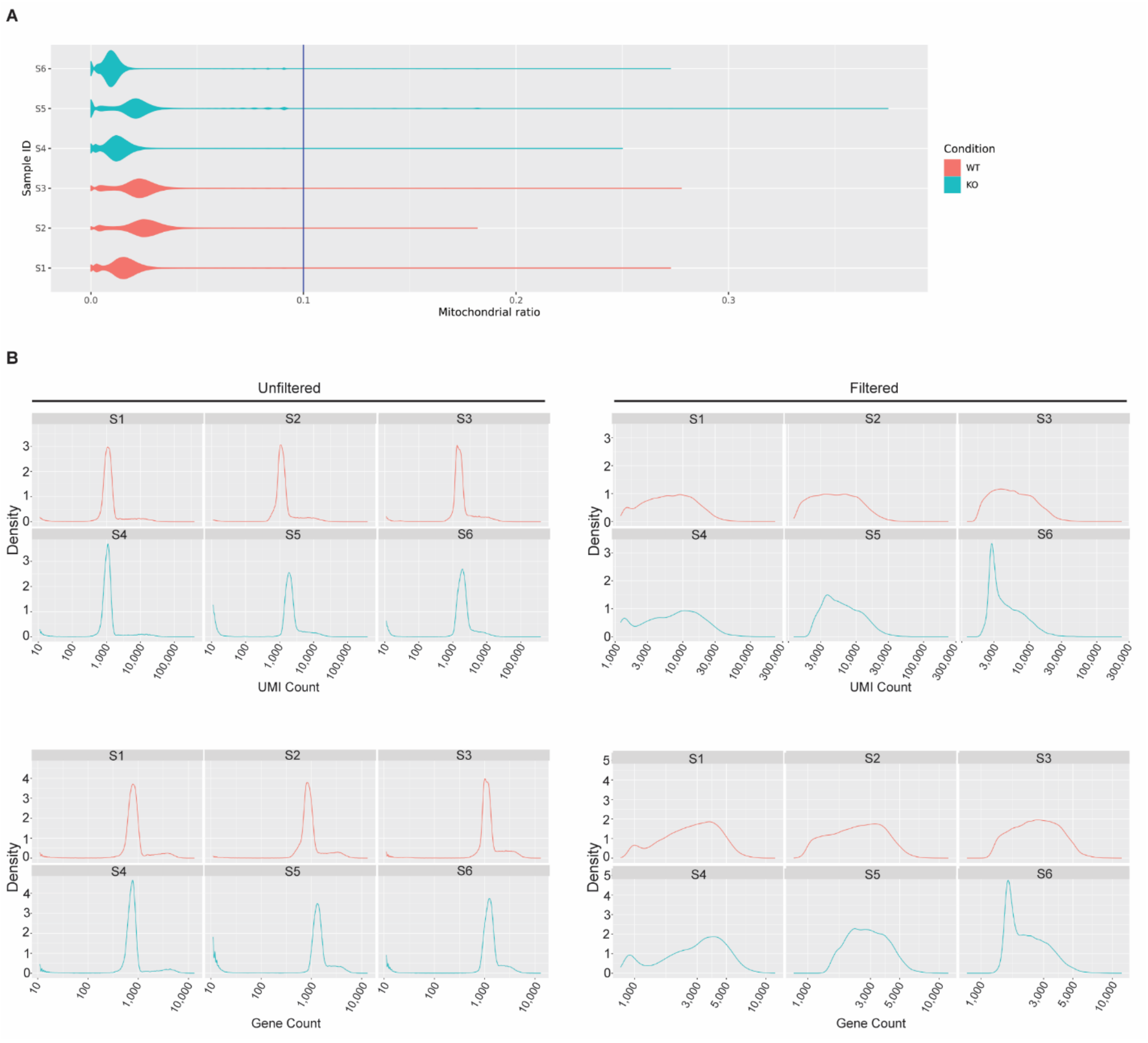

Supplementary Figure 10. Single-nucleus RNA-seq filtering. Single-nucleus RNA-seq was performed on the forebrains of P16 WT (samples 1-3) and *Npc1*$^{-/-}$ (samples 4-6) mice. (A) Nuclei containing a mitochondrial gene proportion greater than ten percent (indicated by blue line) were removed. (B) Nuclei were also filtered out based on their UMI and Gene counts.

Supplementary Table 1. Cell abundance. Number of cells and relative abundance of each cluster in WT and *Npc1*$^{-/-}$ samples. Statistics determined by differential abundance analysis.

Cell Cluster	WT Raw Cell Counts	*Npc1*$^{-/-}$ Raw Cell Counts	WT Percent of Total	*Npc1*$^{-/-}$ Percent of Total	T Statistic	P-Value	FDR
Astrocyte	5865	4627	14.875	11.731	-2.09	0.077	0.299
Endothelial Cell	1568	1487	3.977	3.770	-0.252	0.81	0.929
Excitatory Neuron 1	6139	9690	15.570	24.568	1.459	0.195	0.468
Excitatory Neuron 2	7546	7176	19.138	18.192	-0.328	0.753	0.929
Immature Oligodendrocyte	1244	616	3.155	1.562	-5.669	0.001***	0.005
Inhibitory Neuron 1	3048	2975	7.730	7.543	-0.02	0.985	0.985
Inhibitory Neuron 2	6008	5718	15.238	14.497	-0.378	0.717	0.929
Mature Oligodendrocytes	1325	338	3.360	0.857	-9.51	>0.0001****	0
Mixed	1326	1716	3.363	4.351	0.466	0.658	0.929
Monocyte/Microglia	1489	1739	3.776	4.409	0.941	0.38	0.759
OPC	2121	1626	5.379	4.123	-1.912	0.1	0.299
Pericyte	1750	1734	4.438	4.396	-0.195	0.851	0.929

Supplementary Table 2. Upregulated neuronal genes with decreased H3K27me3.

Gene	Log_2(FC_RNA)	Log_2(FC_H3K27me3)	Log_2(FC_H3K27ac)
Sort1	0.688593981	-0.493917455	2.139628959
Lhfpl4	0.870386038	-0.425246718	1.484033592
Kcna6	0.430819834	-0.567468121	0.559415945
Map6	0.340267701	-0.433326696	0.584399379
Hcn1	0.729944281	-0.348369627	No peak detected
Gng13	1.523419574	-0.497405096	No peak detected
Cnih2	0.457425421	-0.350703492	No peak detected
Pvalb	1.644017944	-0.363215345	No peak detected
Dpysl5	0.633186094	-0.317717436	0.674751049
Brsk2	0.286436989	-0.317340825	1.059961956
Hapln2	1.363540368	-0.464463212	2.507656889
Lrp4	0.492743284	-0.329061633	1.290703836
Kcnip2	0.703757024	-0.407241657	No peak detected
Synpr	0.905858246	-0.716593962	0.905140814
Cnih3	0.882593686	-0.383561338	No peak detected
Cpeb1	1.630711119	-0.47649965	1.52354976
