# Supplementary material for: The cholesterol transporter NPC1 is essential for epigenetic regulation and maturation of oligodendrocyte lineage cells
Source: Nat Commun. 2023 Jul 5;14:3964. doi: 10.1038/s41467-023-39733-6 (PMC10322873; doi:10.1038/s41467-023-39733-6)
Supplement: Supplementary file 3 — Description of Additional Supplementary Files [file 41467_2023_39733_MOESM3_ESM.pdf]

Description of additional supplementary files

Supplementary Data 1 : Differentially expressed genes for the oligodendrocyt e lineage from snRNA-seq. Fold change is expression in Npc1-/- cells relative to WT.

Supplementary Data 2 : GO analysis in oligodendrocyt e lineage cells. GO: Biological Process was performed on the differentially expressed genes detected by snRNA-seq.
