# Supplementary material for: The cholesterol transporter NPC1 is essential for epigenetic regulation and maturation of oligodendrocyte lineage cells
Source: Nat Commun. 2023 Jul 5;14:3964. doi: 10.1038/s41467-023-39733-6 (PMC10322873; doi:10.1038/s41467-023-39733-6)
Supplement: Supplementary file 6 — Reporting Summary [file 41467_2023_39733_MOESM6_ESM.pdf]

nature portfolio

Corresponding author(s): Andrew P. Lieberman; Daniel A. Jacobson

Last updated by author(s): Jun 1, 2023

Reporting Summary

Nature Portfolio wishes to improve the reproducibility of the work that we publish. This form provides structure for consistency and transparency in reporting. For further information on Nature Portfolio policies, see our Editorial Policies and the Editorial Policy Checklist.

Statistics

For all statistical analyses, confirm that the following items are present in the figure legend, table legend, main text, or Methods section.

n/a	Confirmed	
☐	☒	The exact sample size (*n*) for each experimental group/condition, given as a discrete number and unit of measurement
☐	☒	A statement on whether measurements were taken from distinct samples or whether the same sample was measured repeatedly
☐	☒	The statistical test(s) used AND whether they are one- or two-sided<br>*Only common tests should be described solely by name; describe more complex techniques in the Methods section.*
☐	☒	A description of all covariates tested
☐	☒	A description of any assumptions or corrections, such as tests of normality and adjustment for multiple comparisons
☐	☒	A full description of the statistical parameters including central tendency (e.g. means) or other basic estimates (e.g. regression coefficient) AND variation (e.g. standard deviation) or associated estimates of uncertainty (e.g. confidence intervals)
☐	☒	For null hypothesis testing, the test statistic (e.g. *F*, *t*, *r*) with confidence intervals, effect sizes, degrees of freedom and *P* value noted<br>*Give P values as exact values whenever suitable.*
☒	☐	For Bayesian analysis, information on the choice of priors and Markov chain Monte Carlo settings
☒	☐	For hierarchical and complex designs, identification of the appropriate level for tests and full reporting of outcomes
☒	☐	Estimates of effect sizes (e.g. Cohen's *d*, Pearson's *r*), indicating how they were calculated

Our web collection on statistics for biologists contains articles on many of the points above.

Software and code

Policy information about availability of computer code

Data collection	Whole brain saggittal sections were captured using ZEN 2 v2.0.0.0. Confocal images were captured using NIS-Elements AR v5.02.01. qRT-PCR data was gathered using 7500 Software v2.3. Western blot images were captured using iBright software v1.7.0.
Data analysis	The following were used in the processing and analysis of bulk RNA-seq data: BCL Convert Conversion Software v3.9.3 (Illumina), FastQC v0.11.9, MultiQC v1.12, STAR aligner v2.7.9a, MultiQC v1.12, R v4.1.1.<br>The following were used in the processing and analysis of snRNA-seq data: STARsolo aligner v2.7.9a, MultiQC v1.12, Seurat v4.1.0, R v4.1.1, ggplot2 v3.3.6<br>The following were used in the processing and analysis of ChIP-seq data: MACS2 v2.2.7.1, SICER v1.1, ngsplot v2.61, IGV v2.15.1<br>GraphPad Prism 9.0 was used for all t-test and ANOVA analyses.<br>ImageJ 1.52q was used in measuring western blot band intensity and in measuring g-ratios from TEM images. CellProfiler 4.0.1 was used to quantify cell counts and staining intensity in immunofluorescent images.

For manuscripts utilizing custom algorithms or software that are central to the research but not yet described in published literature, software must be made available to editors and reviewers. We strongly encourage code deposition in a community repository (e.g. GitHub). See the Nature Portfolio guidelines for submitting code & software for further information.

Data

Policy information about availability of data

All manuscripts must include a data availability statement. This statement should provide the following information, where applicable:

- Accession codes, unique identifiers, or web links for publicly available datasets
- A description of any restrictions on data availability
- For clinical datasets or third party data, please ensure that the statement adheres to our policy

The data and materials supporting the findings of this study are available from the corresponding authors upon reasonable request. The raw ChIP-seq, snRNA-seq, and bulk RNA-seq data have been submitted deposited into the GEO database under the series accession code GSE221610 [https://www.ncbi.nlm.nih.gov/geo/query/acc.cgi?acc=GSE221610]. Sequence alignment was done using the mouse reference genome GRCm38 [https://www.ncbi.nlm.nih.gov/assembly/GCF_000001635.20/]. Source Data is included with this manuscript.

Human research participants

Policy information about studies involving human research participants and Sex and Gender in Research.

Reporting on sex and gender	N/A
Population characteristics	N/A
Recruitment	N/A
Ethics oversight	N/A

Note that full information on the approval of the study protocol must also be provided in the manuscript.

Field-specific reporting

Please select the one below that is the best fit for your research. If you are not sure, read the appropriate sections before making your selection.

☒ Life sciences ☐ Behavioural & social sciences ☐ Ecological, evolutionary & environmental sciences

For a reference copy of the document with all sections, see nature.com/documents/nr-reporting-summary-flat.pdf

Life sciences study design

All studies must disclose on these points even when the disclosure is negative.

Sample size	Sample size was determined using power analysis.
Data exclusions	no data exclusions were made.
Replication	Due to restrictions in sample availability, only 1 replicate of cells pooled from 6 mice per genotype was used for ChIP-seq experiments. bulk RNA-seq, snRNA-seq, and TEM imaging were done with 3 biological replicates. Some experiments using Cre mouse lines were done with n's of 4 due to mouse availability. All other experiments were done with 5 biological replicates. Data were consistent across replicates.
Randomization	Mice were genotyped prior to being randomly assigned to a treatment group.
Blinding	Mouse studies were carried out by a single individual and were therefore not blinded. Because of this, unbiased pipelines were used whenever possible during data analysis (e.g. CellProfiler).

Reporting for specific materials, systems and methods

We require information from authors about some types of materials, experimental systems and methods used in many studies. Here, indicate whether each material, system or method listed is relevant to your study. If you are not sure if a list item applies to your research, read the appropriate section before selecting a response.

Materials & experimental systems

n/a	Involved in the study
☐	☒ Antibodies
☒	☐ Eukaryotic cell lines
☒	☐ Palaeontology and archaeology
☐	☒ Animals and other organisms
☒	☐ Clinical data
☒	☐ Dual use research of concern

Methods

n/a	Involved in the study
☐	☒ ChIP-seq
☒	☐ Flow cytometry
☒	☐ MRI-based neuroimaging

Antibodies

Antibodies used

The following primary antibodies (antigen [clone], dilution, vendor, cat. no.) were used for these studies: NPC1 [EPR5209], 1:500, Abcam, ab134113; NeuN [A60], 1:500, MilliporeSigma, MAB377; Ki67 [polyclonal], 1:200, Abcam, ab15580; Vinculin [hVIN-1], 1:2000, MilliporeSigma, V9131; Neurofilament [N52], 1:500, MilliporeSigma, MAB5266; H3K27me3 [C36B11], 1:200 [IF], 1:1000 [WB], Cell Signaling Technologies, 9733S; H3K9me3 [D4W1U], 1:200 [IF], 1:1000 [WB], Cell Signaling Technologies, 13969S; Histone H3 [96C10], 1:2000, Cell Signaling Technologies, 3638S; MBP [12], 1:100 [IF], 1:500 [WB], Abcam, ab7349; OLIG2 [polyclonal], 1:500 [IF], 1:1000 [WB], MilliporeSigma, AB9610; SOX10 [SP267], 1:400, Abcam, ab227680; SOX10 [A-2], 1:200, Santa Cruz Biotechnology, sc-365692; H3K27me3 [polyclonal], ActiveMotif, cat. #39155; H3K27ac [polyclonal], ActiveMotif, cat. #39133.

The following secondary antibodies (antigen, dilution, vendor, cat. no.) were used: goat anti-rabbit IgG (H+L)-HRP conjugate, 1:2000, Bio-Rad, 1706515; goat anti-mouse IgG (H+L)-HRP conjugate, 1:2000, Bio-Rad, 1706516; anti-rat IgG HRP conjugated, 1:2000, R&D Systems, HAF005; Alexa Fluor 488 goat anti-rat IgG (H+L), 1:500, Invitrogen, A11006; Alexa Fluor 594 goat anti-rat IgG (H+L), 1:500, Invitrogen, A11007; Alexa Fluor 594 goat anti-mouse IgG (H+L), 1:500, Invitrogen, A11032; Alexa Fluor 488 goat anti–rabbit IgG (H+L), 1:500, Invitrogen, A11008.

Validation

All antibodies are commercially available and have been validated by their manufacturer for use in western blot, ChIP, immunohistochemistry, and/or immunocytochemistry. Our lab has run western blots with the antibodies to confirm band sizes appear at the proper sizes. Cell knockout validation was done when available.

Animals and other research organisms

Policy information about studies involving animals; ARRIVE guidelines recommended for reporting animal research, and Sex and Gender in Research

Laboratory animals

Mice (mus musculus) used in this study were a F1 hybrid resulting from a cross of C57BL6/J and BALB/cJ strains. These mice were used at ages P6, P9, and P16. Smpd1-/- mice were maintained on the C57BL6/J background. Smpd1-/- mice were used at P16. Npc1 flox/flox, Olig2-Cre, and Syn1-Cre mice were also maintained on the C57BL6/J background and used at P16.

Wild animals

No wild animals were used in this study.

Reporting on sex

approximately equal numbers of male and female mice were used, as neurological symptoms has been reported in both sexes in this model.

Field-collected samples

No field-collected samples were used in this study.

Ethics oversight

All animal procedures were approved by the University of Michigan Committee on the Use and Care of Animals (PRO00010017)

Note that full information on the approval of the study protocol must also be provided in the manuscript.

ChIP-seq

Data deposition

☒ Confirm that both raw and final processed data have been deposited in a public database such as GEO.

☒ Confirm that you have deposited or provided access to graph files (e.g. BED files) for the called peaks.

Data access links
May remain private before publication.

To review GEO accession GSE221610:
Go to https://www.ncbi.nlm.nih.gov/geo/query/acc.cgi?acc=GSE221610
Enter token uvavoeqwdjyxbuj into the box

Files in database submission

GSM6764929_3_0D46_01RQUM_WT-IV-VI_H3K27me3_mm_i23.fastq.gz; GSM6764930_4_0D47_01RQUM_NPC-IV-VI_H3K27me3_mm_i33.fastq.gz; GSM6764931_1_0D44_01RQUM_WT-IV-VI_H3K27Ac_mm_i23.fastq.gz; GSM6764932_2_0D45_01RQUM_NPC-IV-VI_H3K27Ac_mm_i25.fastq.gz;
GSM6764929_3_0D46_01RQUM_WT-IV-VI_H3K27me3_mm10_i29_dmnorm_signal.bw;
GSM6764930_4_0D47_01RQUM_NPC-IV-VI_H3K27me3_mm10_i33_dmnorm_signal.bw;
GSM6764931_1_0D44_01RQUM_WT-IV-VI_H3K27Ac_mm10_i23_uniqnorm_signal.bw;

GSM6764932_2_0D45_01RQUM_NPC-IV-VI_H3K27Ac_mm10_i25_uniqnorm_signal.bw;

Genome browser session (e.g. UCSC)	https://tinyurl.com/2lbyodjd

Methodology

Replicates	One replicate for each genotype was used. One replicate contained cells pooled together from six mice.
Sequencing depth	(Sample name; total reads; unique reads (without duplicate reads)).<br>3_0D46_01RQUM_WT-IV-VI_H3K27me3_mm10_i29; 43,383,603; 18,720,302.<br>4_0D47_01RQUM_NPC-IV-VI_H3K27me3_mm10_i33; 42,262,438; 23,376,275.<br>1_0D44_01RQUM_WT-IV-VI_H3K27Ac_mm10_i23; 43,834,918; 16,238,937.<br>2_0D45_01RQUM_NPC-IV-VI_H3K27Ac_mm10_i25; 44,404,991; 15,309,904.<br>All reads are single-end.
Antibodies	H3K27me3 (ActiveMotif, cat. #39155); H3K27ac (ActiveMotif, cat. #39133)
Peak calling parameters	Peak calling was performed by ActiveMotif. The two main peak callers used at Active Motif are MACS/MACS2 (Zhang et al., Genome Biology 2008, 9:R137) and SICER (Zang et al., Bioinformatics 25, 1952-1958, 2009).
Data quality	Only reads that pass Illumina's purity filter, align with no more than 2 mismatches, and map uniquely to the genome are used in analysis. In addition, duplicate reads ("PCR duplicates") are removed.
Software	ChIP-seq analysis was performed by ActiveMotif. The following were used in the processing and analysis of ChIP-seq data: MACS2, SICER, ngsplot v2.61, IGV v2.15.1.
